# Large-scale pattern of genetic differentiation within African rainforest trees: insights on the roles of ecological gradients and past climate changes on the evolution of *Erythrophleum* spp (Fabaceae)

**DOI:** 10.1186/1471-2148-13-195

**Published:** 2013-09-12

**Authors:** Jerome Duminil, Richard P Brown, Eben-Ezer BK Ewédjè, Patrick Mardulyn, Jean-Louis Doucet, Olivier J Hardy

**Affiliations:** 1 Service Evolution Biologique et Ecologie, CP160⁄12, Faculté des Sciences, Université Libre de Bruxelles, 50 Av. F. Roosevelt, 1050 Brussels, Belgium; 2 Bioversity International, Forest Genetic Resources Programme, Sub-Regional Office for Central Africa, P.O. Box 2008 Messa, Yaoundé, Cameroon; 3 School of Natural Sciences and Psychology, Liverpool John Moores University, Liverpool L3 3AF, UK; 4 Faculté des Sciences et Techniques, BP 4521, Université d’Abomey-Calavi, Abomey-Calavi, Benin; 5 Laboratoire de Foresterie des Régions Tropicales et Subtropicales, Unité de Gestion des Ressources Forestières et des Milieux Naturels, Gembloux Agro-Bio Tech, Université de Liège, Passage des Déportés, 5030 Gembloux, Belgium

**Keywords:** Cluster analysis, Fragmentation, Last glacial maximum, Phylogeography, Range expansion, Refugium, Species delimitation, Tropical rainforest

## Abstract

**Background:**

The evolutionary events that have shaped biodiversity patterns in the African rainforests are still poorly documented. Past forest fragmentation and ecological gradients have been advocated as important drivers of genetic differentiation but their respective roles remain unclear. Using nuclear microsatellites (nSSRs) and chloroplast non-coding sequences (pDNA), we characterised the spatial genetic structure of *Erythrophleum* (Fabaceae) forest trees in West and Central Africa (Guinea Region, GR). This widespread genus displays a wide ecological amplitude and taxonomists recognize two forest tree species, *E. ivorense* and *E. suaveolens*, which are difficult to distinguish in the field and often confused.

**Results:**

Bayesian-clustering applied on nSSRs of a blind sample of 648 specimens identified three major gene pools showing no or very limited introgression. They present parapatric distributions correlated to rainfall gradients and forest types. One gene pool is restricted to coastal evergreen forests and corresponds to *E. ivorense*; a second one is found in gallery forests from the dry forest zone of West Africa and North-West Cameroon and corresponds to West-African *E. suaveolens*; the third gene pool occurs in semi-evergreen forests and corresponds to Central African *E. suaveolens*. These gene pools have mostly unique pDNA haplotypes but they do not form reciprocally monophyletic clades. Nevertheless, pDNA molecular dating indicates that the divergence between *E. ivorense* and Central African *E. suaveolens* predates the Pleistocene. Further Bayesian-clustering applied within each major gene pool identified diffuse genetic discontinuities (minor gene pools displaying substantial introgression) at a latitude between 0 and 2°N in Central Africa for both species, and at a longitude between 5° and 8°E for *E. ivorense*. Moreover, we detected evidence of past population declines which are consistent with historical habitat fragmentation induced by Pleistocene climate changes.

**Conclusions:**

Overall, deep genetic differentiation (major gene pools) follows ecological gradients that may be at the origin of speciation, while diffuse differentiation (minor gene pools) are tentatively interpreted as the signature of past forest fragmentation induced by past climate changes.

## Background

**Figure 1:**
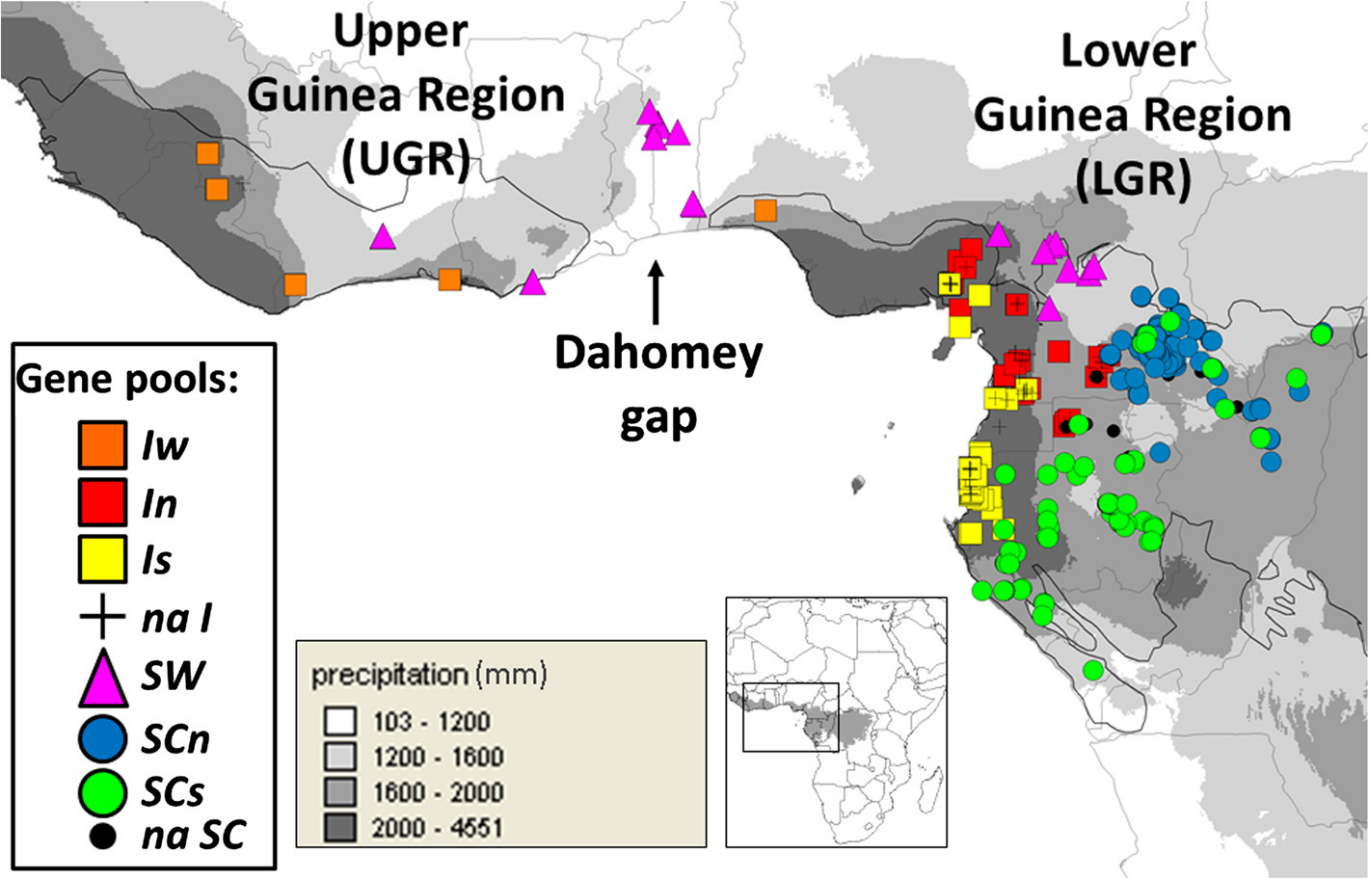
**Sampling of the *Erythrophleum *individuals within the Guinean Region (GR) and assignment to gene pools according to the Bayesian clustering analyses.** The levels of rainfall are indicated on the map, providing the approximate distribution of evergreen (rainfall >2000 mm), semi-evergreen (1600–2000 mm) and dry rainforests (1200–1600 mm). The black line is an approximation of the current distribution of wet rainforests. The distribution of major and minor gene pools as inferred from nSSRs is indicated as follow. Purple triangles: *SW* gene pool, blue and green circles: respectively *SCn* and *SCs* gene pools; orange, red and yellow squares: respectively *Iw*, *In*, *Is* gene pools; crosses (na *I*): *E. ivorense* individuals not attributed to any of minor gene pools *I*; black dots (na *SC*): *E. suaveolens* individuals not attributed to any of minor gene pools *SC*.

The evolution of African rainforest species is still poorly understood. The Guineo-Congolian tropical forests cover a wide area, forming one block extending from Sierra Leone to Ghana in West Africa, and another block from southern Nigeria to the Albertine rift in Central Africa. According to White [1], three main types of Guineo-Congolian rainforests occur along a rainfall gradient displaying a coastal-inland orientation: evergreen forest (“Hygrophilous coastal evergreen rainforest”; rainfall >2000 mm); semi-evergreen forest (“Mixed moist semi-evergreen rainforest”; rainfall between 1600 and 2000 mm); and dry forest (“Drier peripheral semi-evergreen rainforest”; rainfall between 1200 and 1600 mm). Furthermore, the Guineo-Congolian phytochoria has been divided into three sub-centres of endemism along a West–east axis based on patterns of species distribution and endemism [2]. These are: i) the Upper Guinea region (UGR) in West Africa, ii) the Lower Guinea region (LGR) to the West of Central Africa, and iii) the Congolian region to the East of Central Africa. The UGR and LGR are separated by a broad savanna corridor, also known as the Dahomey Gap (Benin, Togo, and South East of Ghana; Figure 1). A range of factors, including spatial ecological gradients, historical climate changes, and physical barriers to gene flow might have contributed to the organization of the diversity within the African rainforest, both within and among species. Given the scarcity of palynological data in the lowland tropics, molecular phylogenetics and population genetics approaches can provide major insights into the relative influence of these factors. The present work aims to provide a better understanding of the evolutionary history of the Guinean tropical forests by addressing the phylogeography of two sister-tropical tree species across the UGR and LGR (hereafter referred to together as the Guinea region: GR).

Genetic studies of plastid and nuclear DNA in plant species from the LGR have already demonstrated that within-species genetic diversity often displays spatial discontinuities leading to geographically coherent gene pools or clades, even in the absence of gaps in the distribution of the species [3-8]. However, the distribution of genetic diversity over the entire GR and the origin of this structuring remain largely unknown. Two main hypotheses may explain these spatial genetic discontinuities: (i) the presence of ecological gradients driving adaptive differentiation (‘ecological gradient hypothesis’), (ii) forest fragmentation/expansion following climatic oscillations driving differentiation by genetic drift between forest refuges (‘forest refuge hypothesis’).

The ecological gradient hypothesis posits that environmental gradients can induce parapatric speciation (or ecotypic differentiation) without the need for population isolation. This hypothesis has been supported in a few African animal species (e.g. [9]) and African plants (e.g. [10]). The forest refuge hypothesis posits that the cyclic fragmentation and re-expansion of lowland forests in response to Pleistocene climatic change has been a main driver for species diversification or population differentiation. The climate was drier in tropical Africa during the last glacial episode (i.e., 110000 to 12000 years BP, with a glacial maximum between 26000 and 19000 years BP) causing an expansion of dry and/or mountain forests and savannas that would have constituted barriers to gene flow among the different rainforest fragments [11,12]. The geographic locations of these refugia are highly debated. Humid conditions had initially returned by *ca.* 14500 years BP, reaching a maximum between 9000 and 6000 years BP. This corresponds to the so-called Holocene African humid period which was characterised by a maximal extension of rainforests [13]. At this time, between *ca.* 8400 and 4500 years BP, even the Dahomey Gap was covered by the rainforest [14]. A significant increase in aridity at the end of the mid-Holocene led to the retreat of the rainforests and the reopening of the Dahomey Gap [15].

*Erythrophleum* (Fabaceae*-*Caesalpinioideae) is a pantropical woody genus with representative species from North-East Asia (*E. fordii, E. succirubrum, E. densiflorum*), Australia (*E. chlorostachys*), Madagascar (*E. couminga*) and Africa (*E. ivorense, E. suaveolens, E. africanum*). In this study we focus on the two recognized African forest species: *Erythrophleum ivorense* A. Chev. (syn. *E. micranthum* Harms) and *E. suaveolens* (Guill. et Perr.) Brenan (syn. *E. guineense* G. Don.). *E. ivorense* occurs in the UGR and the LGR, from Gambia to Gabon, with a distribution discontinuity between the UGR and the LGR, being totally absent from the Dahomey Gap [16]. This species is found in evergreen forests and requires high rainfall [8,17,18]. *E. suaveolens* is more widespread, being found from Senegal to Sudan and Kenya and southward to Mozambique and Zimbabwe [16,17]. Hence, it is present in all three sub-centres of endemism of the Guineo-Congolian phytogeographic region and also occurs in adjacent peri-forest areas. It occupies a wider range of climates than *E. ivorense* and is found in semi-evergreen forests (in Central Africa; see [8]) and in gallery forests (outside of Central Africa; [16,17,19]). Contrary to *E. ivorense*, its distribution is continuous from West to Central Africa, occurring also in the Dahomey Gap [16]. The two species are difficult to distinguish in the field in the absence of reproductive organs, and are grouped under the same timber trade name “tali”. In Central Africa, the literature contains several inconsistencies concerning their respective distributions and ecological requirements. Nevertheless, Duminil *et al.*[8] demonstrated that plastid DNA (pDNA) represents an efficient barcode to identify the two species in the LGR where their respective parapatric distributions are correlated with the rainfall gradient. In West Africa, the two species are reported to occur in contrasting habitats (*E. ivorense* in evergreen forests and *E. suaveolens* in forest galleries) and are not expected to be found in sympatry [17]. A third related African species, *E. africanum* (Welw. ex Benth.) Harms, is distributed within the Zambezian and Sudanian regional centres of endemism (*sensu* White [2]), from Senegal to Sudan, Kenya and Tanzania and southward to the Transvaal region, occurring in woodland savannas but never in forests. Nuclear DNA sequences indicate that African *Erythrophleum* species form a clade in which *E. ivorense* and *E. suaveolens* are more related to each other than to *E. africanum* (J. Duminil, unpublished).

The wide ecological amplitude and geographic distribution of *E. ivorense* and *E. suaveolens* within the GR allow analysis of the influence of ecological gradients and past population fragmentation on patterns of genetic differentiation. As taxonomists describe two species which are often confused (at least in Central Africa), we hypothesize that genetic variation might display diffuse discontinuities. Sharp discontinuities (i.e. occurrence of gene pools without introgression) would be expected between isolated reproductive entities, like species following the Biological Species Concept or populations that have been geographically isolated over a long period. Diffuse genetic discontinuities (i.e. gene pools with evidence of substantial introgression) would occur between populations that were (i) recently separated (e.g. by the formation of the Dahomey gap) or (ii) formerly isolated (e.g. in forest refugia) but subsequently came into secondary contact or (iii) parapatric, and adapted to different environments but still maintain some gene flow, in which case they may form a stable hybrid zone.

Here we use nSSRs and pDNA sequences to (i) identify major gene pools (sharp discontinuities) and minor gene pools (diffuse discontinuities) within the GR region and check whether they can be matched to taxonomic species, and (ii) verify whether genetic discontinuities can be explained by current climatic gradients (ecological gradient hypothesis) and/or by Pleistocene climatic changes (forest refuge hypothesis and role of the Dahomey Gap). We attempt to gain complementary insights into the evolution of *Erythrophleum* (i) by assessing the congruence between gene pools assessed from biparentally-inherited nSSRs and maternally-inherited pDNA haplotypes and clades, (ii) by estimating the divergence times between gene pools using a fossil-calibrated pDNA phylogenetic tree, and (iii) by testing for genetic signature of past demographic changes using nSSRs.

## Methods

### Sampling and DNA extraction

Leaves or cambium were sampled from 648 individuals of *E. ivorense* and *E. suaveolens* from the LGR and the UGR (*sensu* White [2]). As most samples could not be identified taxonomically using morphological traits, except samples from Central Africa [8], they were treated as a blind sample for genetic analyses.

Correspondence with taxonomic species is based, in Central Africa, on a previous published work [8]. In West Africa, we considered *a priori* that samples from the evergreen forest area (>2000mm rainfall, Figure 1) must belong to *E. ivorense* while those from the dry forest area (<1600mm rainfall) belong to *E. suaveolens*, in accordance with the known ecology of the species in West Africa [17]. For each sample, the annual precipitation of the sampling location was recovered from WorldClim (variable Bio12).

Additional *E. africanum* samples from Benin (N10.21 E1.21 and N10.68 E1.63), *E. chlorostachys* (F.Muell.) Baill. from Australia (S12.49 E130.99) and *E. fordii* Oliv. from China (three individuals, one from Fujian—N24.1 E117.4—, one from Guangdong—N23.6 E114.7—, one from Guangxi—N22.1 E108.3—) were used as outgroups in phylogenetic analyses. Total DNA was isolated with the NucleoSpin^®^ plant kit (Macherey-Nagel).

### Microsatellite genotyping and diversity indices

Nine microsatellite loci (namely Ery1, Ery3, Ery4, Ery6, Ery7, Ery14, Ery 17, Ery18 and Ery23) were amplified for 648 individuals of *E. ivorense* or *E. suaveolens* using the protocol described in [20].

As the concept of a population is difficult to apply in tropical rainforests, individuals were arbitrarily grouped into 34 populations according to their geographic coordinates. Observed and expected heterozygosities, inbreeding coefficient (*F*_IS_) and allelic richness (A_O_) were estimated for each population using FSTAT [21]. The frequency of null alleles per locus and per population was estimated in the 18 populations represented by at least 10 individuals by maximum likelihood following [22] using the program FREENA [23].

### Identifying genetic discontinuities

We used the Bayesian assignment program STRUCTURE [24] to identify differentiated gene pools without prior spatial information on the origin of samples. We used a two-step approach to identify (i) major gene pools showing no or negligible introgression (sharp boundaries) and (ii) minor gene pools displaying introgression (diffuse boundaries) within each major gene pool. Hence, analyses were first run on the whole set of nSSR genotypes using the no admixture and independent allele frequencies model, to avoid overestimation of *K*[25] and because gene pools having diverged for long may have independent allele frequencies. The admixture model was also tested and gave similar results (data not shown). There were 20 independent iterations for each imposed number of gene pools *K* varying from 1 to 10, which were summarized using CLUMPP v.1.1.2 [26]. The total MCMC chain length was 40000 steps with the first 10000 steps being discarded as burn-in [27]. To assess the optimal value of *K* we considered the log-likelihood of the data according to *K*, but also the number of individuals that were not assigned to a gene pool above a probability threshold of 0.8. The latter criterion was important to identify well isolated gene pools (few or no admixed individuals).

Further STRUCTURE analyses were carried out within each major gene pool using the same procedure except that we used an admixture model with correlated allele frequencies, in which the fraction of ancestry from each gene pool was estimated for each individual. A uniform prior was used whereby the individual admixture alpha parameter was set to be equal for all gene pools. Here, the optimal *K* was assessed by the log-likelihood of the data only.

### Patterns of genetic differentiation

Fixation indices (*F*_ST_ and *R*_ST_) between pairs of populations or gene pools were computed using SPAGeDi 1.3 [28]. *F*_ST_ is based on allele identity while *R*_ST_ is an analogue of *F*_ST_ based on allele size. *R*_ST_ is expected to be larger than *F*_ST_ if stepwise mutations have contributed to differentiation, as in the case of ancient isolation [29]. The occurrence of this pattern was tested using random permutations of allele sizes among allelic states [29].

### pDNA genetic diversity distribution

We characterized the genetic variation at the trnC-petN1R inter-genic fragment, as described in [8], on a sub-set of 297 individuals representative of all defined populations (Additional file 1).

We also sequenced the *matK* gene and the *trnL* intron using the matK1R-matK3F and trnLc-trnLd primer pairs (respectively Ki-Joong Kim, unpublished; and [30]). Sequences were obtained for 72 (*matK*) and 66 individuals (*trnL*) arbitrarily selected from the different gene pools defined by the Bayesian clustering analyses described above. All three pDNA fragments were also sequenced for *E. africanum*, *E. chlorostachys* and *E. fordii*.

### Phylogenetic network reconstruction

A median joining network [31] for the *trnC*-*petN1R* inter-genic fragment was constructed using the software NETWORK version 4.5.1.6 (Fluxus Technology Ltd). All polymorphic characters were considered (single nucleotide polymorphism, insertion-deletion and simple sequence repeat) except for a small hypervariable portion of the *trnC*-*petN1R* inter-genic fragment that could not be unambiguously aligned. All characters were given equal weight. Samples of *E. africanum* were used to root the network (unambiguous nucleotide sequence alignment was not possible for *E. chlorostachys* or *E. fordii*, and these were discarded from the analysis).

### Molecular dating analyses

The *matK* gene and the *trnL* intron were used in the dating analyses because they have already been used by Bruneau *et al.*[32] to date a phylogenetic tree of Fabaceae species, with calibration points specified from fossil dates. Here we focused on the part of the phylogenetic tree that includes *Erythrophleum* (Figure four in [32]). We obtained *trnL* and *matK* sequences from GenBank for a set of Fabaceae species closely related to *Erythrophleum*, and attempted to obtain examples from the most divergent lineages within the *Erythrophleum* clade (Figure four in [32]). For *E. ivorense* and *E. suaveolens* sequences, we used a single sample per major gene pool.

**Figure 2:**
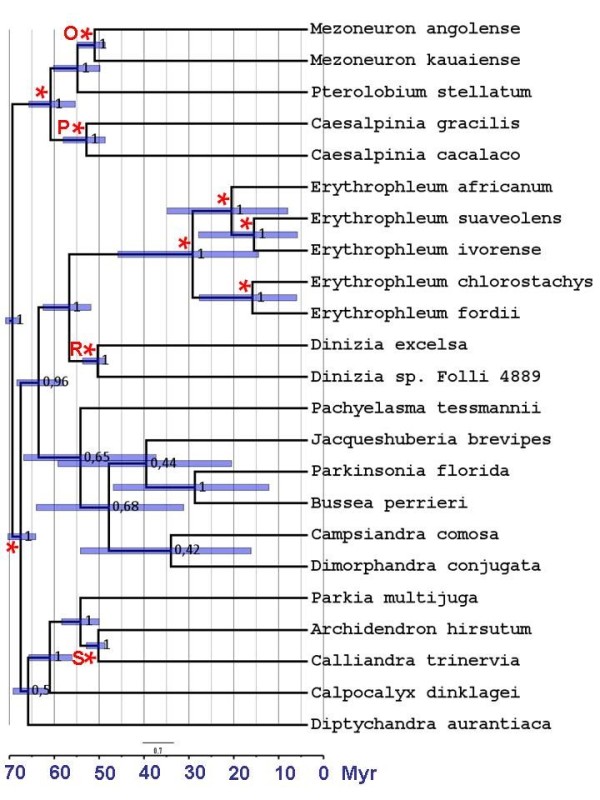
**Divergence time chronogram of the Bayesian maximum clade credibility tree based on the chloroplast fragments *matK *and *trnL*.** The blue bars represent the 95% highest posterior density intervals for each divergence time. Fossil calibration nodes are indicated by letters (P, O, R and S) as in [32]. Nodes constrained to be monophyletic are indicated by *. Numbers at internal nodes represent clades posterior probabilities.

The phylogeny and divergence times were estimated using the Bayesian MCMC software package BEASTv1.6.1 [33]. The HKY (for *trnL*) and the GTR (for each of the three *matK* codon positions) models of DNA substitution were used with a gamma distribution for among site rate variation (these were the best supported models under the AIC criterion in jModelTest [34]). We constrained some taxa to be monophyletic following unequivocal results in a previous study [32] (see Figure 2).

Rates were specified from the uncorrelated lognormal relaxed molecular clock and a birth-death model was used to specify the prior on times. Prior information on divergence times was obtained from a previous review of fossil-information (paleobotanical records) [31] and used to specify calibrations on specific nodes. Given the age of the root, and the scale dependence of some components of the model, it was reasonable to define 1 time unit = 10 Ma (rather than 1 Ma). Priors on four node ages (nodes O, P, R and S; see below for their designation) were specified from the Normal distribution, N (4.5,1), which was asymmetrically truncated by the uniform distribution, U (4.5, 5.6). The minimum value of this constraint was justified because there is fossil evidence that divergence within all of these clades began > 45 Ma, reviewed in [31]. Also, the estimated divergence times at more basal nodes than O, P, R and S are estimated to be around 56 Ma. Specifically, earliest divergence time within the *Caesalpinia* clade is 55.9 Ma [31], while the earliest divergence time for nodes R and S must be younger than the most recent common ancestor (MRCA) of the *Dimomorpha*, *Mimosoideae*, *Tachigali*, and *Peltophorum* clades which has been estimated at 56.3 Ma [31]. To summarize, our analysis constrained the ages of the following four nodes to be between 45 and 56 Ma, with greatest prior density around 45 Ma and lowest density around 56 Ma: (1) the most recent common ancestor (MRCA) of the *Caesalpinia* clade (node P in [31]), (2) the MRCA of the *Mezoneuron* clade (node O), (3) the MRCA of the *Dinizia* clade (node R), (4) the MRCA of the *Ingeae* clade (node S). In addition, we followed [31] by constraining the root node (equivalent to the most basal divergence within the legumes) to be close to 65 Ma, using a gamma distribution. We examined the effects of three different Gamma priors placed on the root: G (65, 0.1), G (650, 0.01) and G (6500, 0.001). The first of these represents the loosest calibration and the last represents the tightest: 95% of the G (65, 0.1) distributions lies within the range 52 – 79 Ma, while 95% of the G (6500, 0.001) lies within 63 – 66 Ma.

Two MCMC analyses were run each for 100 million generations sampled at 5000 step intervals. We used Tracer v1.4 [33] to assess convergence, estimate effective sample sizes (ESS), and examine the posteriors of all parameters. The first 500 samples were discarded as burn-in.

### Detection of past variation in population size

Microsatellite markers were used to detect signals of demographic decline or expansion using the Bayesian inferential method of Storz and Beaumont [35] implemented in MSVAR1.3. The method relies on the full allelic distribution and takes into account relative sizes of microsatellite alleles, with mutation rates and demographic parameters free to vary among loci. Allelic frequencies are used to estimate the current effective population size (N_0_), the ancestral effective population size (N_1_), the number of generations since the demographic change (Ta) and the mutation rate (μ). In separate analyses, both *a priori* defined populations (Additional files 2 and 3) and gene pools inferred from Bayesian cluster analyses were used as input to MSVAR. It was important to test demographic changes at both these levels because departure from the Wright-Fisher assumption used in the MSVAR analytical model can create false signals [36]. We used only populations that contained at least 20 individuals, therefore restricting this analysis to the LGR (populations 1, 3, 4, 5, 15, 16, 17, 18, 21 and 25; Additional files 2 and 3). Analyses at the gene pool level were performed only on gene pools presenting a low genetic substructure (see results) to avoid potential population structure bias [36]. We used very flexible priors in order to minimize their impact on the posterior [37]. All priors were specified from log Normal distributions as follows: N_0_ (4, 6), N_1_ (4, 6), μ (-4, 3) and T_a_ (3, 6) (the first value represents the mean and the second value the standard deviation). Priors on the current and the ancestral population size were identical so that population decline or expansion is equally likely. An exponential model of population size change was assumed. The generation time was assumed to be 100 years (modal flowering age based on our field observations, data not shown). Three analyses with different starting points were carried out for each data set (1.2. × 10^9^ steps; thinning of 40000 steps). Convergence among MCMC chains was checked by applying the Gelam-Rubin statistic implemented in the CODA package in R [38]. Samples from the three analyses were combined. The significance of changes in population size was examined using Bayes factors. The number of states in the chains in which the population has declined (N_0_/N_1_ < 1) was divided by the number of states in which the population has expanded (N_0_/N_1_ > 1) (see [37]). The means and the 95% highest probability densities (HPD) of the marginal posteriors of the natural parameters (N_0_, N_1_, μ and T_a_) and the scaled parameters (θ_0_ = 4 N_0 _μ, θ_1_ = 4 N_1_ μ and t_f _ = Ta /2N_0_) were obtained using Tracer v1.4.

## Results

### Bayesian clustering analyses – identification of major gene pools (sharp genetic discontinuities)

The likelihood of the data increased steadily from *K* = 1 up to *K* = 5 under the no-admixture model. A plateau was reached at *K* = 5, above which the variance among replicates increased (Additional file 4). Only a few individuals remained unassigned to a gene pool for both *K* = 2 (10 individuals, at a cut-off level of P ≥ 0.8) and *K* = 3, (12 individuals), while the number of unassigned individuals increased sharply for *K* = 4 (102 individuals) and 5 (419 individuals) (Additional files 5 and 6). Hence, we considered that *K* = 3 is the optimal solution for identifying major gene pools. These gene pools present characteristic geographic distributions (Figure 1 and Additional file 5). A first gene pool is distributed along the African littoral, but is absent from the Dahomey Gap and southern Central Africa regions. This distribution tracks the distribution of evergreen forests. A second gene pool extends from the drier zones of West Africa (including the Dahomey Gap) to the north-west of Cameroon. The third gene pool is restricted to the interior forests of Central Africa, reaching the littoral only in the south of Central Africa. It is noteworthy that the first two gene pools cluster together when two clusters are specified in the analysis (*K* = 2) (Additional file 5).

All (but one) of the Central African *E. ivorense* samples (following [8]) and all West African samples that were originally assigned to *E. ivorense* (15 samples collected in the evergreen forest) were grouped within the first gene pool, which was denoted with the letter “*I*” (Table 1). All West African samples *a priori* belonging to *E. suaveolens* (28 samples collected in the dry forest area) as well as samples from northwest Cameroon identified as *E. suaveolens*[8] were assigned to the second gene pool, which was therefore denoted “*SW*” (Table 1). Finally, all central African samples identified as *E. suaveolens*, except individuals from northwest Cameroon included in *SW*, were assigned to the third gene pool, which was therefore denoted “*SC*”.

**Table 1:** **Correspondence between the three major gene pools and species based on the following proxies: in West Africa, samples were assigned to *E. ivorense *if rainfall >2000 mm and to *E. suaveolens *if rainfall <1600 mm at their sampling location; in Central Africa, samples were assigned to *E. ivorense *if they had the plastid haplotype H1, and to *E. suaveolens *if they had another haplotype, following **[8]

	**Major gene pools (*K*** **= 3)**				
**Species**	***SW***	***SC***	***I***	**Unassigned**	**Total**
West Africa					
*E. ivorense*	0	0	15	0	15
*E. suaveolens*	27	0	0	0	27
Unassigned	1	0	9	0	10
Total	28	0	24	0	52
Central Africa					
*E. ivorense*	0	1	96	0	97
*E. suaveolens*	9	142	0	2	153
Unassigned	3	252	81	10	346
Total	12	395	177	12	596

### Bayesian clustering analyses – identification of minor gene pools (diffuse genetic discontinuities)

Using the admixture model within each primary gene pool, the optimal *K* reached *K* = 3 within *I*, *K* = 1 within *SW*, *K* = 2 within *SC*. Hence, a total of six minor gene pools can be identified (Figure 1). They again formed geographically cohesive but more or less overlapping groups and a substantial number of individuals now appears admixed. According to their geographic distributions, the three minor gene pools detected within *I* will be referred to as *Iw* (found in UGR and on the north-western part of the LGR, Figure 1), *In* (found in the northern part of the LGR) and *Is* (found in the southern part of the LGR). The two minor gene pools detected within *SC* will be referred to as *SCn* (northern LGR) and *SCs* (southern LGR) (Figure 1, Additional file 7)*.* At a threshold of 80%, approximately 40% of *I* individuals could not be assigned to *Iw*, *In* or *Is*, and approximately 20% of *SC* individuals could not be assigned to *SCn* or *SCs* (Additional file 7).

### Genetic diversity analyses

Analyses used either the arbitrary-defined populations (local patterns of diversity) or the six minor gene pools (regional patterns of diversity) as the unit of classification. At the population level, significant departures from Hardy-Weinberg equilibrium were detected (excess of homozygotes). Mean null allele frequencies per population varied from 0.005 to 0.181. Estimated selfing rate was close to zero in all but two populations. The two exceptions were populations 3 and 12, although these were not characterized by higher *F*_IS_ (Additional file 2). Mean allelic richness varied from 1.33 to 10.70.

At the gene pool level, the *F*_ST_ among populations differed significantly from zero in all but one gene pool (*Is*), but were always lower than 0.147. Permutation tests on *R*_ST_ revealed no evidence of a phylogeographic signal in any of the gene pools except for *Is* (Table 2).

**Table 2:** Population-level genetic diversity and differentiation at nSSRs within each minor gene pool

**Gene pool **^**A**^	**N. populations**	***N ind***	***A***_**O**_^**B**^	***H***_**O**_^**C**^	***H***_**S**_^**D**^	***H***_**T**_^**E**^	***F***_**ST**_^**F**^	***R***_**ST**_^**G**^
*SCn*	9	305	3.24 ± 1.08	0.522	0.616	0.631	0.016*	0.002 ^NS^
*SCs*	6	95	3.26 ± 1.02	0.513	0.673	0.684	0.008 ^NS^	0.062*
*SW*	5	40	NA ^I^	0.542	0.598	0.651	0.099*	0.174 ^NS^
*In*	6	136	3.60 ± 0.79	0.423	0.726	0.749	0.043*	0.082 ^NS^
*Is*^H^	3	46	NA ^I^	0.435	0.586	0.605	0.056*	0.034 ^NS^
*Iw*^H^	3	20	3.49 ± 1.04	0.426	0.645	0.745	0.147*	-0.009 ^NS^

^A ^see Figure 1 for the designation of the gene pools; ^B ^Allelic richness; ^C^ Observed proportion of heterozygotes; ^D^ Within population gene diversity; ^E^ Overall gene diversity; ^F^ * indicates that the population differentiation index (*F*_ST_) is significantly different from 0 as measured by an exact G test_;_^G^ * indicates that *R*_ST_ is significantly superior to the *F*_ST_ (presence of a phylogeographic signal); ^H^ Locus 14H was excluded (high frequencies of null alleles) in all the analyses for this gene pool; ^I^ Not available due to missing data for one locus in at least one of the population.

Overall, five of the 15 pairwise comparisons between the minor gene pools resulted in a significant phylogeographic signal (*R*_ST_ significantly larger than *F*_ST_, Table 3). These significant comparisons were generally population pairs from different major gene pools within the UGR and the LGR (*Iw*-*SCs*, *SW*-*SCn*, *SW*-*SCs*). However a significant comparison was found within a major gene pool (*Iw*-*Is*), as well between two major gene pools in the LGR (*Is*-*SCs*).

**Table 3:** **Pairwise genetic differentiation between minor gene pools estimated by *F***_**ST **_**(lower diagonal matrix) and *R***_**ST **_**(upper diagonal matrix)**

	***SW***	***SCn***	***SCs***	***Is***	***In***	***Iw***
***SW***		0.342*	0.381*	0.146	0.149	0.197
***SCn***	0.223		0.050	0.321	0.218	0.157
***SCs***	0.198	0.040		0.286*	0.206	0.253*
***Is***	0.169	0.247	0.220		0.117	0.296*
***In***	0.181	0.206	0.189	0.085		0.100
***Iw***	0.190	0.215	0.160	0.100	0.080	

*Test of *R*_ST_ > *F*_ST_ statistically significant at *P* < 0.05.

### pDNA genetic diversity distribution and correspondence with nuclear gene pools

**Figure 3:**
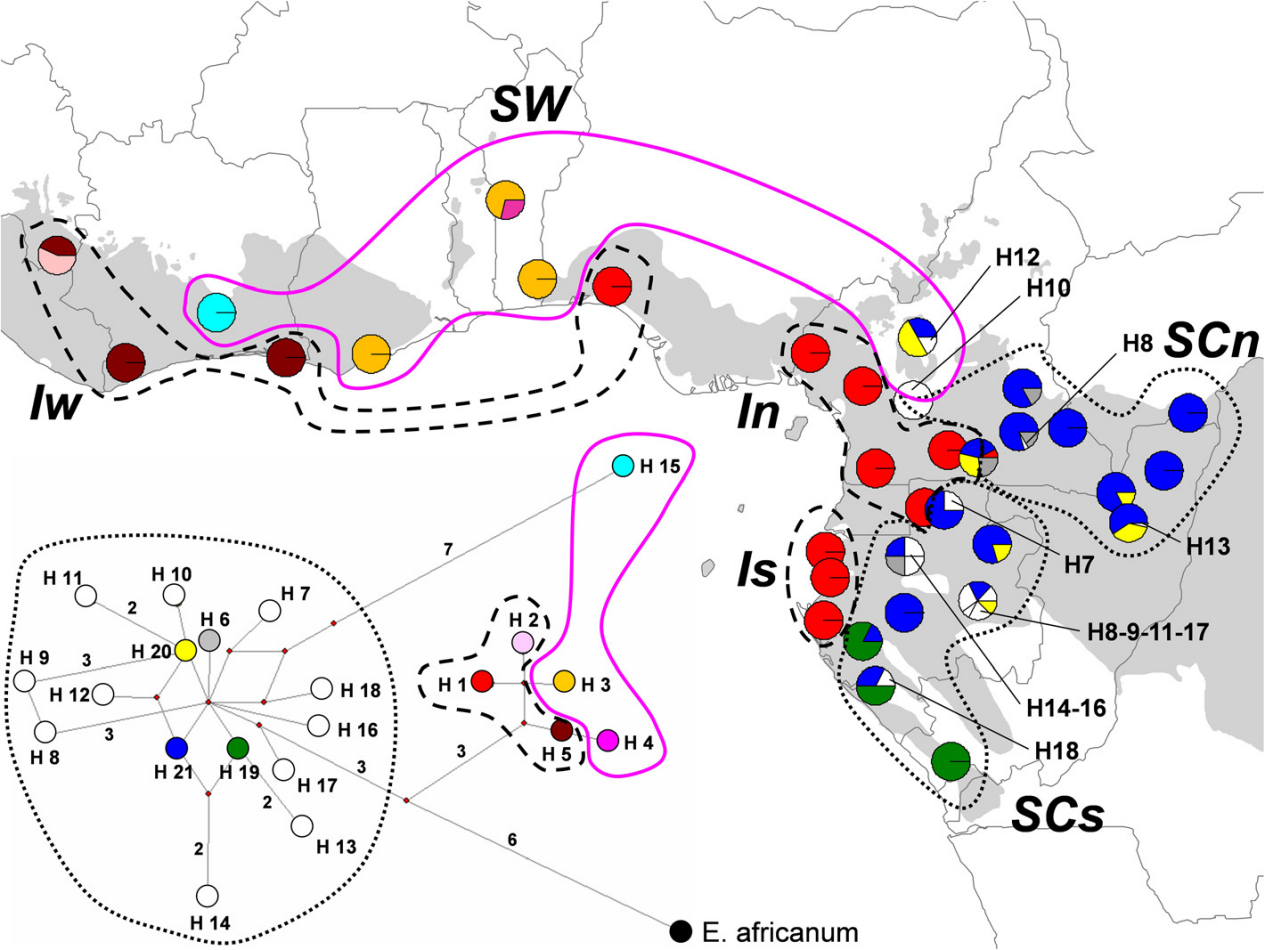
**Distribution and median-joining network of haplotypes of the pDNA inter-genic fragment *trnC*-*petN1R*, and delimitation of the distribution of major and minor gene pools as inferred from nSSRs (pink line: *SW *gene pool, dashed line: *SCn *and *SCs *gene pools; dotted lines: *Iw*, *In*, *Is *gene pools).** Rare haplotypes are indicated directly on the map by a number. Small dots on the haplotype network indicate ancestral nodes or unsampled haplotypes. Branch lengths are proportional to the number of mutations (numbers on branches where > 1 mutation). Haplotypes associated with each of the three major gene pools are encircled on the haplotype network, except that haplotypes H12, H20 and H21, most typical of *SC*, are also found in *SW* populations from Northern Cameroon.

Among the 297 sequences of the *trnC*-*petN1R* inter-genic fragment, 21 different haplotypes were identified (Figure 3, Additional file 8). A first clade of five related haplotypes (H1 to H5) was found along the coast of the LGR (H1), in Benin (H3 and H4), Ghana (H3), Guinea (H2 and H5), and the South of Ivory Coast (H5). H1, H2 and H5 were found only in *I*, with H1 in *In* and *Is*, and H1, H2 and H5 in *Iw*, except one individual from southern Cameroon bearing H1 while unambiguously assigned to *SCn* (population 18, Additional files 2 and 3). H3 and H4 occurred in *SW*. The second clade includes 15 haplotypes (H6 to H21) which were found almost entirely in *SC*. There were two exceptions to this rule: H15 was found in a population from Ivory Coast (population 28, Additional files 2 and 3) from *SW* gene pool; H12, H20 and H21 were found in northwest Cameroon (population 8, Additional files 2 and 3) from *SW* gene pool too. Hence, within the major gene pool *I*, the parapatric minor gene pools *In* and *Is* were monomorphic for the same haplotype (H1) while the allopatric minor gene pool *Iw* also contained H1 East of the Dahomey gap (individuals located in Nigeria) and two other related haplotypes (H2 and H5) found only West of the Dahomey gap. Within the major gene pool *SC*, the parapatric *SCn* and *SCs* shared many haplotypes from the second clade, but with higher haplotype diversity in *SCs* (12 haplotypes) than in *SCn* (6 haplotypes), despite a larger sample size in the later. Gene pool *SW* had mostly unique haplotypes from the first clade (H3, H4) or the second clade (H12, H15) as well as haplotypes shared with gene pool *SC* in their contact zone from North Cameroon (H20, H21).

Eight different haplotypes were characterized in the 72 sequences of the *matK* gene (Additional files 8 and 1). There was no clear genetic structuring over the Guinea region within *I* (the haplotype H4 is represented in the UGR and the LGR) or over the LGR within *SC*. Five haplotypes were detected among the 66 *trnL* sequences (Additional files 8 and 1). No polymorphism was found within *I* or within *SCn*. The geographic patterns of nucleo-cytoplasmic associations were consistent with those reported for the *trnC*-*petN1R* (Additional file 1). Levels of genetic polymorphism and differentiation were generally too low to allow estimation of divergence time between gene pools by molecular dating analyses, except between the *SC* and *I*. Accession numbers corresponding to the *trnC*, *matK* and *trnL* sequences are indicated in the Additional file 8.

### Molecular dating analyses

Posterior mean divergence times and 95% posterior intervals are shown in Figure 2 and Table 4. The posterior mean age of the MRCA was 11.87 Ma between *E. ivorense* (gene pool *I*) and Central African *E. suaveolens* (gene pool *SC*), 16.92 Ma between *E. africanum* and the common ancestor to *E. ivorense* and *E. suaveolens*, and 25.52 Ma for all *Erythrophleum* species.

**Table 4:** **Divergence time estimates of diversification events in *Erythrophleum***

	**Mean divergence time**	**HPD95%**
African / non-African *Erythrophleum*	25.52	[10.74 – 42.13]
*E. chlorostachys* / *E. fordii*	12.17	[2.40 – 24.11]
*E. africanum* / (*E. Ivorense* - *E. suaveolens*)	16.92	[4.36 – 31.39]
*E. suaveolens* / *E. ivorense*	11.87	[2.17 – 24.18]

### Demographic changes

Evidence of demographic decline was detected by MSVAR analyses at both geographic scales (population and gene pool, Additional file 9). The data contained insufficient information to allow precise estimation of natural parameters N_0_, N_1_, Ta and μ. Marginal posterior distributions were wide and differed only slightly from prior distributions. By contrast, the scaled parameters θ_1_ and t_f_ were well estimated with a narrow 95% HPD (except for populations 17 and 21 and for *SCs* and *In* that presented wide 95% HPDs), but not θ_0_ (except for populations 4, 16, 18 and for *SCn*). A signal of population decline was detected in all populations and gene pools, mostly with strong support (Bayes Factor > 10 in 10 cases, although 3 < BF < 10 in one case, and 0.33 < BF < 3, in three cases). Weak or no support corresponded to populations and gene pools in which the data were not very informative about scaled parameters.

## Discussion

### Major gene pools and species delimitation

In the present study, two major gene pools were expected, as samples came from two different species according to taxonomists. We unexpectedly detected three major gene pools, one corresponding to *E. ivorense* (gene pool *I*) and two corresponding to *E. suaveolens* (gene pools *SW* and *SC*; Figure 1). Moreover, the order of appearance of the gene pools (with increasing values of *K*) was not consistent with the expected species delimitation because *I* and *SW* clustered together against *SC* when we fixed *K* = 2. This questions the monophyly of *E. suaveolens* and thus the botanical classification based on morphological characters.

Gene pools *I* and *SC* present a long contact zone in Central Africa, so that the sharp genetic boundary indicates that they should correspond to distinct species following the BSC. The same can be argued between *I* and *SW* in West Africa. By contrast, *SW* and *SC* have a limited contact zone in northern Cameroon, but our limited sample size there does not allow us to confirm they are reproductively isolated. Additional sampling at their contact zone and a new taxonomic study based on morphological traits of *E. suaveolens* individuals from Central and West Africa would be justified to assess whether they might be considered as distinct morphological (sub-) species. Interestingly, significant phylogeographic signals (nSSRs, *R*_ST_ > *F*_ST_) were mostly detected between major gene pools, suggesting ancient divergence (number of generations comparable or larger than the reciprocal of the mutation rate; [23]). This is in support of ongoing or accomplished speciation with a (near) complete barrier to gene flow.

The chloroplast genome was globally congruent with the nSSRs data in terms of haplotype distribution because each major gene pool generally carried private haplotypes. There are two exceptions however: in southern Cameroon, one individual from the *SC* gene pool carried a haplotype typical of the *I* gene pool, and in northern Cameroon, individuals belonging to the *SW* gene pool had some of the haplotypes found in the *SC* gene pool. These exceptions may result from past chloroplast captures or from incomplete lineage sorting. While incomplete lineage sorting can often impede reciprocal monophyly between fully isolated sister species with large population sizes, it is much less likely to maintain shared haplotypes and, moreover, it is unlikely to maintain a geographic correlation in the distribution of shared haplotypes between species. As all the reported exceptions are located close to contact zones between major gene pools, chloroplast capture appears a more parsimonious explanation. It suggests that individuals from the major gene pools might hybridize on rare occasion.

Examination of the distribution of pDNA lineages in each major gene pool reveals some additional incongruences because even the haplotypes unique to *SW* are paraphyletic. Ancient chloroplast captures may also be responsible for this pattern, but incomplete lineage sorting is equally plausible. In any case, this supports rejection of the pDNA-based species delimitation based on the monophyly of pDNA lineages [8].

### Patterns of differentiation within the *Erythrophleum* genus in Africa: ecological gradient versus forest refuge hypothesis

The three major gene pools are found in different habitats and climatic conditions. This is particularly true in West Africa where gene pool *Iw* was found in evergreen forests in locations where annual rainfall ranged from 1620 to 2350 mm (mean = 1980 mm), while gene pool *SW* was found in dry forests and in forest galleries with an annual rainfall ranging from 1055 to 1220 mm (mean = 1150 mm). These results are fully consistent with the West African literature reporting that *E. ivorense* occurs only in wet evergreen forests while *E. suaveolens* occurs in much drier areas. In Central Africa, the contrast is less sharp: gene pools *Is* and *In* were found in evergreen forests with very high annual rainfall (1620 to 3480 mm; mean = 2460 mm), while *SC* occurred in the drier semi-evergreen forests (annual rainfall 1370 to 2050 mm; mean = 1640 mm), and *SW* in the forest-savanna mosaic of northern Cameroon (annual rainfall 1530 to 2090 mm; mean = 1710 mm). Our data do not allow testing whether diversification has been driven by an ecological factor such as annual water availability, length of the dry season or another correlated ecological factor. However, given the above evidence and the fact that the third African species, *E. africanum* is only found in drier savanna habitats than *E. suaveolens*, we can conclude that there is a strong association between species diversification and habitat specialisation in African *Erythrophleum*, suggesting that the differentiation of major gene pools can be explained by the ecological gradient hypothesis.

Contrary to major gene pools, the diffuse genetic boundaries separating minor gene pools were not related to rainfall gradient, or any other obvious environmental gradient. There is thus no support for an ecological gradient hypothesis, in which the forms represent differentiated ecotypes which maintain some degree of gene flow.

Based on pDNA, Central African *E. suaveolens* (gene pool *SC*) and *E. ivorense* (*I*) diverged before the Pleistocene, during the Miocene or the Pliocene (Table 4). As, we examined a deep phylogeny, it seems reasonable to assume that sequence divergence times should closely approximate to species divergence times. Although posteriors are quite wide they support the view that genus diversification of African rainforest plants mostly preceded the Pleistocene glacial-interglacial cycles [39]. Evidence for the impact of Pleistocene climate changes may be present in intraspecific patterns of genetic diversity, but the polymorphism of the pDNA sequences was too low to date the divergence between major gene pools, and the polyphyletic nature of the chloroplast genome within gene pool *SW* precludes any estimation of its divergence time.

### The origin of minor gene pools: population fragmentation and evidence of population decline support the forest refuge hypothesis

Molecular dating did not allow estimation of divergence times between minor gene pools. However, they should be much inferior to the divergence time between major gene pools, and are thus likely to be contemporaneous of the Pleistocene climatic cycles. The *R*_ST_ values provide insights on the relative divergence. Indeed, *Iw* presents a significant phylogeographic signal with respect to *Is*. Differentiation should thus be much more ancient than the differentiation between *In* and *Is*, or between *SCn* and *SCs*, where minor gene pools are in direct contact and there is a high degree of admixture.

Additional insights come from tests of demographic changes. Indeed, we detected a signal of population decline (nSSR data) in all the LGR gene pools, both at the population and gene pool levels, which confirms previous results obtained with pDNA data [8]. This signal is consistent with the forest refuge hypothesis. However, as it could not be accurately dated, we cannot state with certainty that this was driven by climate changes.

Several studies of tree species have reported patterns of genetic differentiation between the northern and the southern part of the LGR with a disjunction located approximately between 0°N and 2°N, despite the current absence of a habitat discontinuity [5-7,40]. We also found this pattern of differentiation with a limit close to 2°N, for both *Erythrophleum* species. Although different factors can generate similar patterns, this convergence among several forest tree species suggests ancient habitat fragmentation, consistent with the forest refuge hypothesis, if we assume the existence of northern and southern refuges. The weak genetic differentiation between the northern and southern gene pools, the absence of phylogeographic signal, and the large proportion of admixed individuals near the contact zone indicate that the LGR minor gene pools within each species are only weakly differentiated and have already partly introgressed.

### The relevance of the Dahomey Gap for the differentiation of rainforest species

The Dahomey Gap is often considered as the limit between Upper and Lower Guinean phytogeographic regions. However, it does not coincide with the delimitation of *SW* and *Iw* gene pools (Figures 1 and 3). This result is not surprising for *E. suaveolens* given that, in West Africa, it mainly occurs in gallery forests within dry forest or savanna landscapes at the periphery of the Guineo-Congolian forest. However, for *E. ivorense*, which is strictly associated with evergreen forests with high rainfall, the distribution of *Iw* on each part of the Dahomey gap is more surprising. In fact, for the nuclear genome, a genetic discontinuity occurs West of the Dahomey gap, in Nigeria (limit between gene pools *Iw* and *In*), while it occurs at the height of the Dahomey gap for the plastid genome (distribution limit of haplotypes H1 and H5; Figure 3). This pattern supports the hypothesis that populations on each part of the Dahomey Gap were recently in contact and that the savanna corridor reopened recently. Meanwhile, the absence of shared pDNA haplotypes between the LGR *Iw* and the UGR *Iw* suggests that the Dahomey gap did play a role in the past. It has been suggested that the Dahomey Gap was dominated by savanna during the glacial periods and by rainforest during the last interglacial and the early Holocene [12,14]. The current savanna corridor was almost certainly present *ca.* 1100 years ago [14] but in southern Benin a semi-evergreen rainforest prevailed between *ca.* 8400 and 4500 years BP [14]. Thus, the distribution of *Iw* on each part of the Dahomey gap and the absence of shared pDNA haplotypes between UGR and LGR populations indicate that the populations were separated in the past during a cooling event. They underwent secondary contact during a warming event and separated again during the last opening of the Dahomey Gap.

The discrepancy between the positions of the nuclear and plastid genetic discontinuities could be explained by more limited seed than pollen dispersal and a phenomenon of chloroplast capture when populations west of the Dahomey gap (*Iw*) extended to the east during the more humid periods and hybridized with eastern populations bearing the H1 haplotype. Additional sampling in Nigeria would be necessary to test this hypothesis. Interestingly, the limit between *Iw* and *In* is localized somewhere between 5° and 8° E (Nigeria), a biogeographic limit coinciding with the one found in lowland forest bird taxa [41].

## Conclusion

Our study provides new insights into patterns of differentiation that occurred within evergreen, semi-evergreen and forest galleries of the Guinea region using a widespread tree genus. We detected three major gene pools occurring in distinct habitat types that probably originated before the Pleistocene. Hence, climatic gradients in tropical Africa are probably the first major driver of differentiation in the genus *Erythrophleum*, but additional tests based on molecular signatures of climate-driven selection would be necessary to confirm this hypothesis. Furthermore, within major gene pools, we provide evidence of historical barriers to gene flow (i) within the evergreen and semi-evergreen forests of the LGR at a latitude between 0 and 2°N, (ii) within the evergreen forest of southern Nigeria at a longitude between 5 and 8° E. The first barrier has been reported in other African rainforest tree species and could indicate the occurrence of Northern and Southern forest refuges in the LGR. The forest refuge hypothesis is also supported by genetic signatures of past population declines. Contrary to *a priori* expectations, the second barrier does not coincide with the Dahomey gap, but to the east of this region, indicating that this savanna corridor was previously dominated by an evergreen forest containing *E. ivorense*. Nevertheless, the occurrence of an east–west genetic discontinuity indicates that West African and Central African forests have probably been isolated most of the time during the Pleistocene. Finally, while three gene pools with sharp genetic boundaries have been detected, chloroplast captures seem to have occurred in a few instances at their contact zones, suggesting that they may not be fully isolated.

### Data accessibility

DNA sequences: accessions numbers provided in Additional file 8.

## Competing interests

The authors declare that they have no competing interests.

## Authors' contributions

The research interests of JD, E-EBKE, J-LD and OJH are primarily in studying the evolution of tree species from tropical African rainforests. RPB is primarily interested in statistical aspects of evolutionary genetics and in examining how major physical events have influenced speciation and local patterns of diversity, particularly in reptiles. PM has a general interest in the analysis of DNA sequence variation for studying evolution, with current research focused mainly on comparing multi-locus phylogeographic patterns among different temperate and cold-adapted herbivorous insects. All authors read and approved the final manuscript.

## Supplementary Material

Additional file 1 **Spatial distribution of the genetic diversity observed for *matK *(above) and for *trnL *(below).** Both figures show the median joining network for *E. suaveolens* and *E. ivorense* rooted on *E. africanum*, *E. chlorostachys* and *E. fordii*. Click here for file

Additional file 2 Sampling locations, diversity data, null allele frequencies and selfing rate estimates. Click here for file

Additional file 3 **Delimitation of populations.** This figure represents the spatial distribution of the individuals included in this study and their clustering in populations. Click here for file

Additional file 4 **Likelihood increasing mean according to K and DeltaK Evanno’s method.** These figures represent likelihood increasing mean according to K and DeltaK. Click here for file

Additional file 5 **Identification of major gene pools: spatial distribution of the individuals assigned to each gene pool (at a threshold probability of 80%) with increasing values of K.** These figures represent the probability assignments of each individuals with increasing values of K (from 2 to 5), and the distribution of each cluster on the map of the region. Click here for file

Additional file 6 **Assignment of the individuals to gene pools at a probability threshold of 80% with increasing values of K.** The table provides numerical values of data represented in Additional file 5. Click here for file

Additional file 7 **Map of admixture zone between the Central African Northern and Southern minor gene pools in *E. ivorense *and *E. suaveolens***. Click here for file

Additional file 8 **GenBank accessions numbers of the plastid DNA sequences obtained in this study.** The table provides the accession numbers for all pDNA haplotypes described in this study. Click here for file

Additional file 9 Demographic inferences obtained by MSVAR analyses at a population and gene-pool level. Click here for file
