# Supplementary material for: Large-scale pattern of genetic differentiation within African rainforest trees: insights on the roles of ecological gradients and past climate changes on the evolution of Erythrophleum spp (Fabaceae)
Source: BMC Evol Biol. 2013 Sep 12;13:195. doi: 10.1186/1471-2148-13-195 (PMC3848707; doi:10.1186/1471-2148-13-195)

Additional file 1: Spatial distribution of the genetic diversity observed for *matK* (above) and for *trnL* (below). Both figures show the median joining network for *E. suaveolens* and *E. ivorense* rooted on *E. africanum*, *E. chlorostachys* and *E. fordii*.

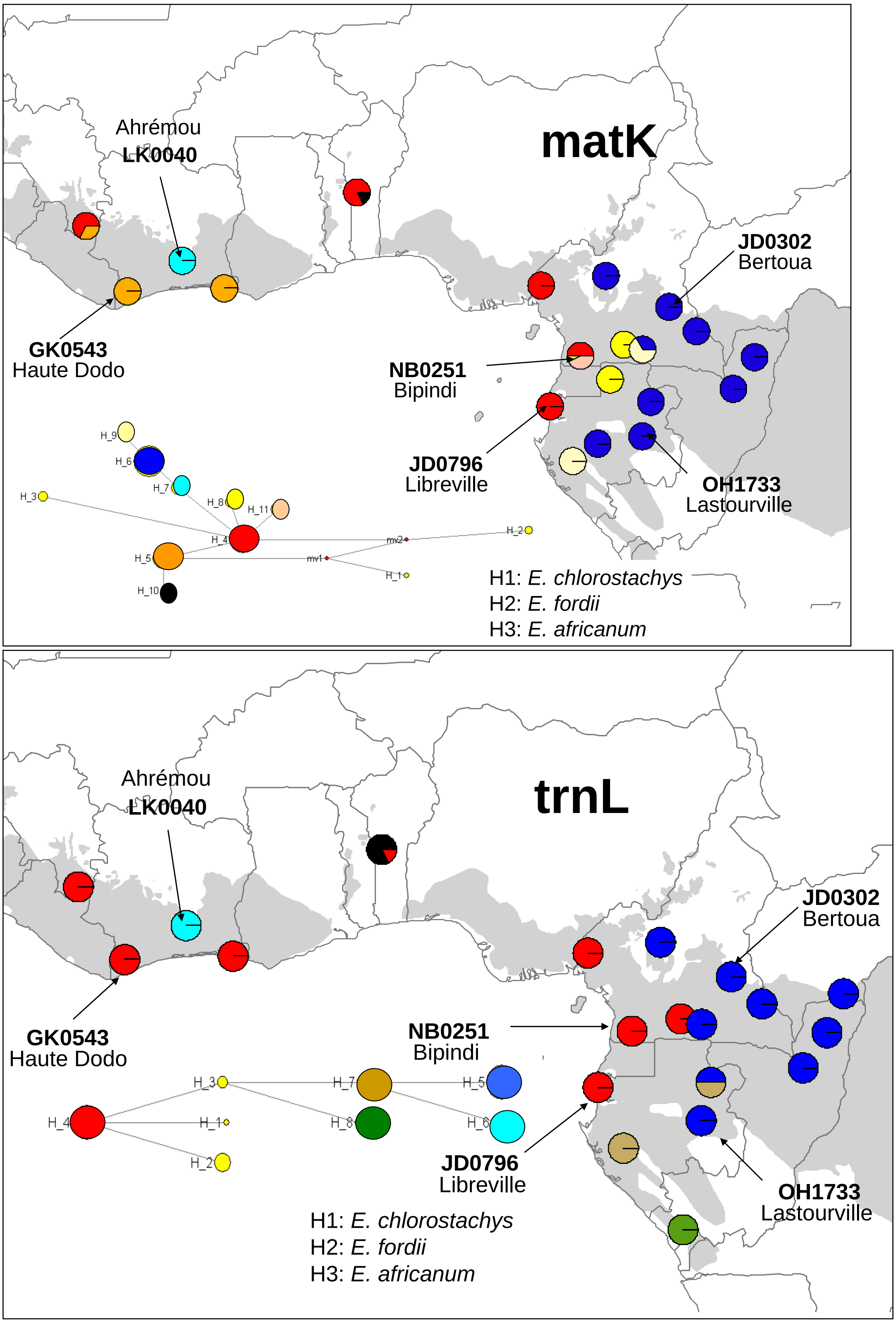

Supplement: Additional file 1 — Spatial distribution of the genetic diversity observed for matK (above) and for trnL (below). Both figures show the median joining network for E. suaveolens and E. ivorense rooted on E. africanum, E. chlorostachys and E. fordii. [file 1471-2148-13-195-S1.pdf]
