# Supplementary material for: Large-scale pattern of genetic differentiation within African rainforest trees: insights on the roles of ecological gradients and past climate changes on the evolution of Erythrophleum spp (Fabaceae)
Source: BMC Evol Biol. 2013 Sep 12;13:195. doi: 10.1186/1471-2148-13-195 (PMC3848707; doi:10.1186/1471-2148-13-195)
Supplement: Additional file 2 — Sampling locations, diversity data, null allele frequencies and selfing rate estimates. [file 1471-2148-13-195-S2.pdf]

Additional file 2: *S*ampling locations, diversity data, null allele frequencies and selfing rate estimates.

No ID	Population name ID	X [A] (long.)	Y [B] (lat.)	Gene pool	Nnr [C]	Ncp [D]	A_O [E]	H_O [F]	H_E [G]	F_{IS} [H]	*null* [I]	$F_{IS(null)}$ [J]
1	**Korup**	9.0213	5.2738	*In*	51	8	8.78	0.522	0.770	0.329	0.129 ± 0.019	0.020
2	**Douala**	10.2466	4.4945	*In*	11	9	5.44	0.399	0.721	0.471	0.158 ± 0.120	0.073
3	**Bipindi**	10.5480	2.5685	*In*	49	32	7.78	0.359	0.678	0.484	0.181 ± 0.141	0.037
4	**Libreville**	9.3727	0.6187	*Is*	23	14	5.25	0.449	0.645	0.314	0.116 ± 0.111	0.035
5	**Pongara**	9.4873	0.0056	*Is*	21	14	5.22	0.457	0.628	0.296	0.089 ± 0.090	0.021
6	**Lambaréné**	9.3309	-1.0011	*Is*	4	2	2.14	0.393	0.418	0.106	--	--
7	**Mayombe**	10.5493	-2.5326	*SCs*	12	6	6.00	0.559	0.683	0.303	0.114 ± 0.098	0.021
8	**Foumban**	11.5380	5.6425	*SCw*	7	6	3.89	0.528	0.696	0.258	--	--
9	**Yaounde1**	11.4384	4.1612	*SCn*	1	1	1.33	0.333	0.333	na	--	--
10	**Yaoundé2**	12.2456	2.9926	*In*	17	9	6.33	0.398	0.702	0.444	0.163 ± 0.123	0.030
11	**Oyem1**	11.7013	1.6501	*In*	10	10	4.22	0.439	0.661	0.363	0.116 ± 0.131	0.022
12	**Cristal**	11.2402	0.5067	*SCs*	5	4	1.71	0.714	0.714	0.267	--	--
13	**Mouila**	10.2509	-1.4879	*SCs*	7	6	5.11	0.496	0.606	0.269	--	--
14	**Lopé**	11.2234	-0.8195	*SCs*	6	5	4.11	0.584	0.589	0.125	--	--
15	**Bertoua**	13.9972	4.4469	*SCn*	26	12	6.67	0.521	0.605	0.142	0.034 ± 0.081	0.021
16	**Mindourou**	13.9128	3.4287	*SCn*	174	25	6.22	0.489	0.691	0.172	0.077 ± 0.018	0.022
17	**Yokadouma**	15.0599	3.5091	*SCn*	21	8	6.67	0.550	0.675	0.189	0.064 ± 0.067	0.016
18	**Bikoula**	12.9682	2.8066	*SCn/In*	21	13	6.22	0.594	0.633	0.063	0.034 ± 0.081	0.010
19	**Oyem2**	12.1591	1.5946	*SCs*	4	4	4.78	0.497	0.667	0.252	--	--
20	**Makokou**	13.2855	0.7922	*SCs*	12	5	4.78	0.611	0.690	0.189	0.068 ± 0.062	0.032
21	**Lastourville**	12.9520	-0.5345	*SCs*	53	15	4.33	0.472	0.620	0.253	0.090 ± 0.088	0.030
22	**Lolé**	17.8880	3.8606	*SCn*	11	8	8.89	0.484	0.645	0.005	0.028 ± 0.059	0.026
23	**Moloundou**	16.1924	2.0091	*SCn*	11	6	10.67	0.544	0.657	0.190	0.060 ± 0.073	0.023
24	**Pokola**	17.3201	2.5135	*SCn*	5	5	2.89	0.426	0.548	0.120	--	--
25	**Yoko**	16.4894	1.2926	*SCn*	32	25	6.22	0.471	0.602	0.221	0.076 ± 0.062	0.027
26	**Mont Nimba**	-8.6648	7.5597	*Iw*	9	7	3.67	0.280	0.539	0.500	--	--
27	**HteDodo**	-7.0535	5.0455	*Iw*	5	4	4.25	0.438	0.695	0.405	--	--
28	**Ahrémou**	-4.9251	6.2212	*SW*	6	4	3.00	0.581	0.541	-0.085	--	--
29	**Mélékoukro**	-3.2940	5.1632	*Iw*	6	5	3.00	0.588	0.551	-0.083	--	--
30	**Ghana**	-1.2912	5.2540	*SW*	6	6	2.22	0.222	0.345	0.378	--	--
31	**Manigri**	1.8637	8.8598	*SW*	14	14	4.67	0.590	0.655	0.090	0.055 ± 0.088	0.020
32	**Adja-Ouéré**	2.6149	7.0077	*SW*	2	2	2.75	0.875	0.854	-0.053	--	--
33	**Omo**	4.3708	6.8359	*Iw*	4	2	2.78	0.435	0.659	0.453	--	--
34	**PointeNoire**	12.3203	-4.3090	*SCs*	1	1	2.33	0.667	0.611	na	--	--

[A] Mean longitude and [B] Latitude of the population in decimal degrees; [C] Number of individuals genotypes for microsatellite markers; [D] Number of individuals sequenced for the chloroplast inter-genic fragment *trnC-petN1R*; A_O [E] Allelic richness; H_O [F] Observed heterozygosity; H_E [G] Expected heterozygosity; [H] F_{IS} Inbreeding coefficient; [I] Mean frequency of null alleles over all loci; [J] Inbreeding coefficient once null allele effect was controlled.
