# Supplementary material for: Large-scale pattern of genetic differentiation within African rainforest trees: insights on the roles of ecological gradients and past climate changes on the evolution of Erythrophleum spp (Fabaceae)
Source: BMC Evol Biol. 2013 Sep 12;13:195. doi: 10.1186/1471-2148-13-195 (PMC3848707; doi:10.1186/1471-2148-13-195)

Additional file 3: Delimitation of populations. The numbers correspond to the ones indicated in Table S2

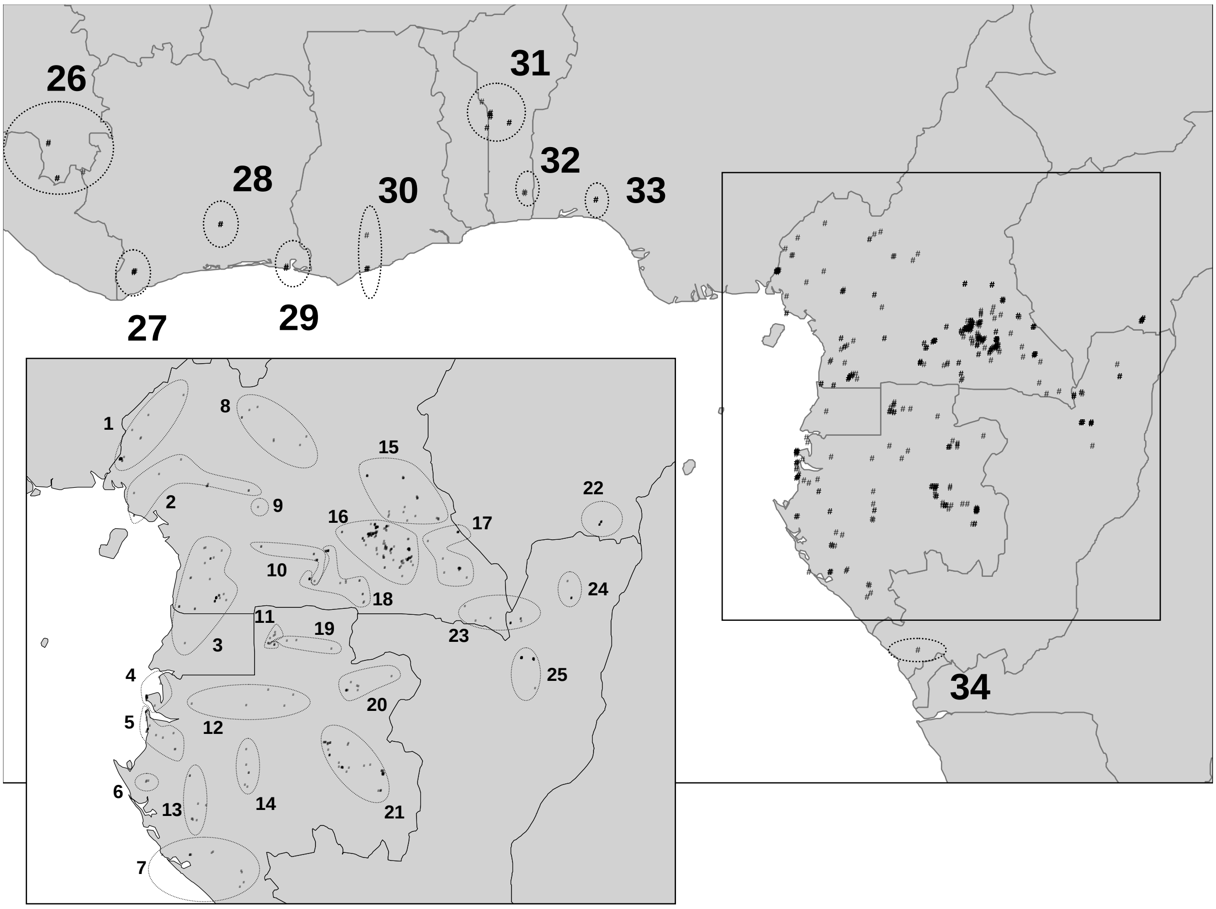

Supplement: Additional file 3 — Delimitation of populations. This figure represents the spatial distribution of the individuals included in this study and their clustering in populations. [file 1471-2148-13-195-S3.pdf]
