# Supplementary material for: Large-scale pattern of genetic differentiation within African rainforest trees: insights on the roles of ecological gradients and past climate changes on the evolution of Erythrophleum spp (Fabaceae)
Source: BMC Evol Biol. 2013 Sep 12;13:195. doi: 10.1186/1471-2148-13-195 (PMC3848707; doi:10.1186/1471-2148-13-195)
Supplement: Additional file 4 — Likelihood increasing mean according to K and DeltaK Evanno’s method. These figures represent likelihood increasing mean according to K and DeltaK. [file 1471-2148-13-195-S4.pdf]

Additional file 4: Likelihood increasing mean according to K and DeltaK Evanno's method. Output were obtained using STRUCTURE HARVESTER (Earl & vonHoldt, 2012)

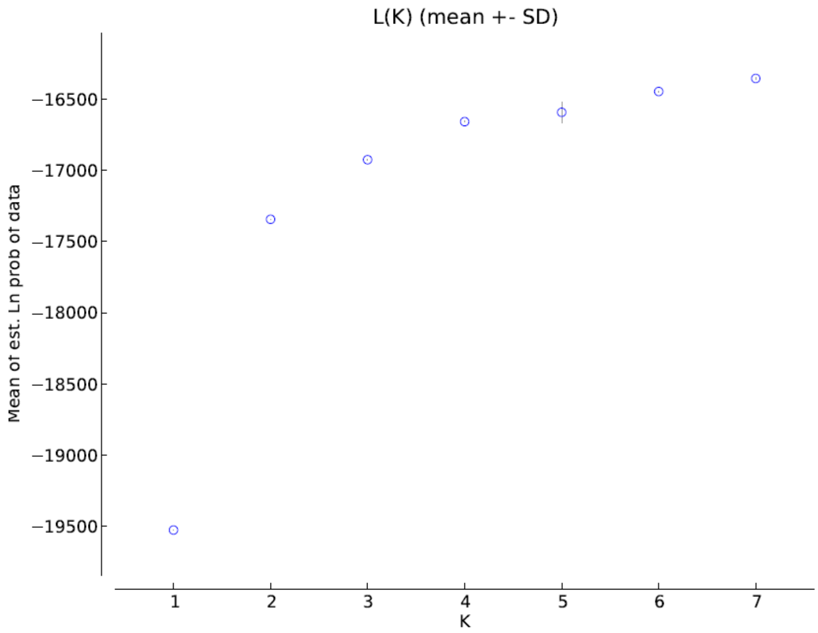

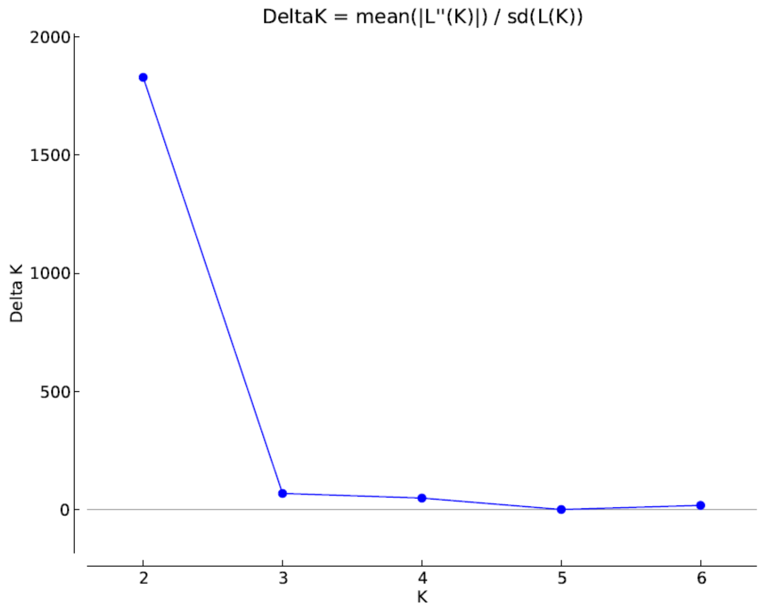

Earl, DA & vonHoldt BM (2012) STRUCTURE HARVESTER: a website and program for visualizing STRUCTURE output and implementing the Evanno method. Conservation Genetics Resources 4(2), 359-361.
