# Supplementary material for: Large-scale pattern of genetic differentiation within African rainforest trees: insights on the roles of ecological gradients and past climate changes on the evolution of Erythrophleum spp (Fabaceae)
Source: BMC Evol Biol. 2013 Sep 12;13:195. doi: 10.1186/1471-2148-13-195 (PMC3848707; doi:10.1186/1471-2148-13-195)

Additional file 5: Identification of major gene pools: spatial distribution of the individuals assigned to each gene pool (at a threshold probability of 80%) with increasing values of K. The barplots represent the probability of assignment to gene pools for each individual. The number of individuals per gene pool is indicated in the additional file 6.

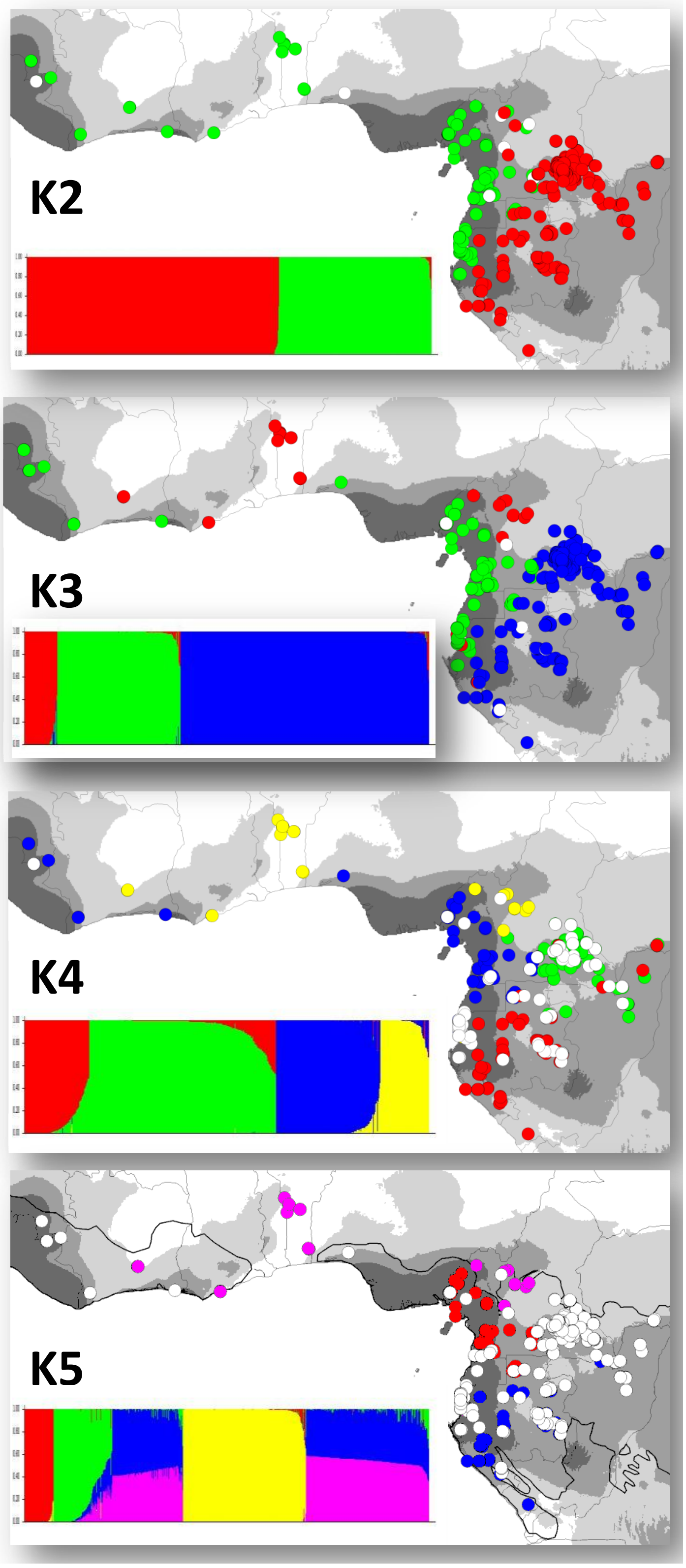

Supplement: Additional file 5 — Identification of major gene pools: spatial distribution of the individuals assigned to each gene pool (at a threshold probability of 80%) with increasing values of K. These figures represent the probability assignments of each individuals with increasing values of K (from 2 to 5), and the distribution of each cluster on the map of the region. [file 1471-2148-13-195-S5.pdf]
