# Supplementary material for: Large-scale pattern of genetic differentiation within African rainforest trees: insights on the roles of ecological gradients and past climate changes on the evolution of Erythrophleum spp (Fabaceae)
Source: BMC Evol Biol. 2013 Sep 12;13:195. doi: 10.1186/1471-2148-13-195 (PMC3848707; doi:10.1186/1471-2148-13-195)
Supplement: Additional file 6 — Assignment of the individuals to gene pools at a probability threshold of 80% with increasing values of K. The table provides numerical values of data represented in Additional file 5. [file 1471-2148-13-195-S6.pdf]

Additional file 6: Assignment of the individuals to gene pools at a probability threshold of 80% with increasing values of K. See additional file 5 for the corresponding spatial distribution of gene pools and for the distribution of assignment probability of individuals to gene pools.

**K =**	Non-assigned individuals	Red dots	Blue dots	Violet dots	Green dots
2	10	236	402		
3	12	194	395	47	
4	102	156	264	42	84
5	419	130	60	39	
