# Supplementary material for: Large-scale pattern of genetic differentiation within African rainforest trees: insights on the roles of ecological gradients and past climate changes on the evolution of Erythrophleum spp (Fabaceae)
Source: BMC Evol Biol. 2013 Sep 12;13:195. doi: 10.1186/1471-2148-13-195 (PMC3848707; doi:10.1186/1471-2148-13-195)

Additional file 7: Map of admixture zone between the Central African Northern and Southern minor gene pools in *E. ivorense* and *E. suaveolens*. *E. ivorense*: red squares correspond to *In*, yellow ones to *Is* and white circles to unassigned individuals (assignment probability to a gene pool always <80%). *E. suaveolens*: blue dots correspond to *SCn*, green dots to *SCs* and black dots to unassigned individuals. The diagrams on the left part and on the right part of the figure represent the probabilities of individual assignment to gene pools respectively for *E. ivorense* and *E. suaveolens*. In each of these diagrams, the northern most individuals are on the upper part and the southern most on the lower part.

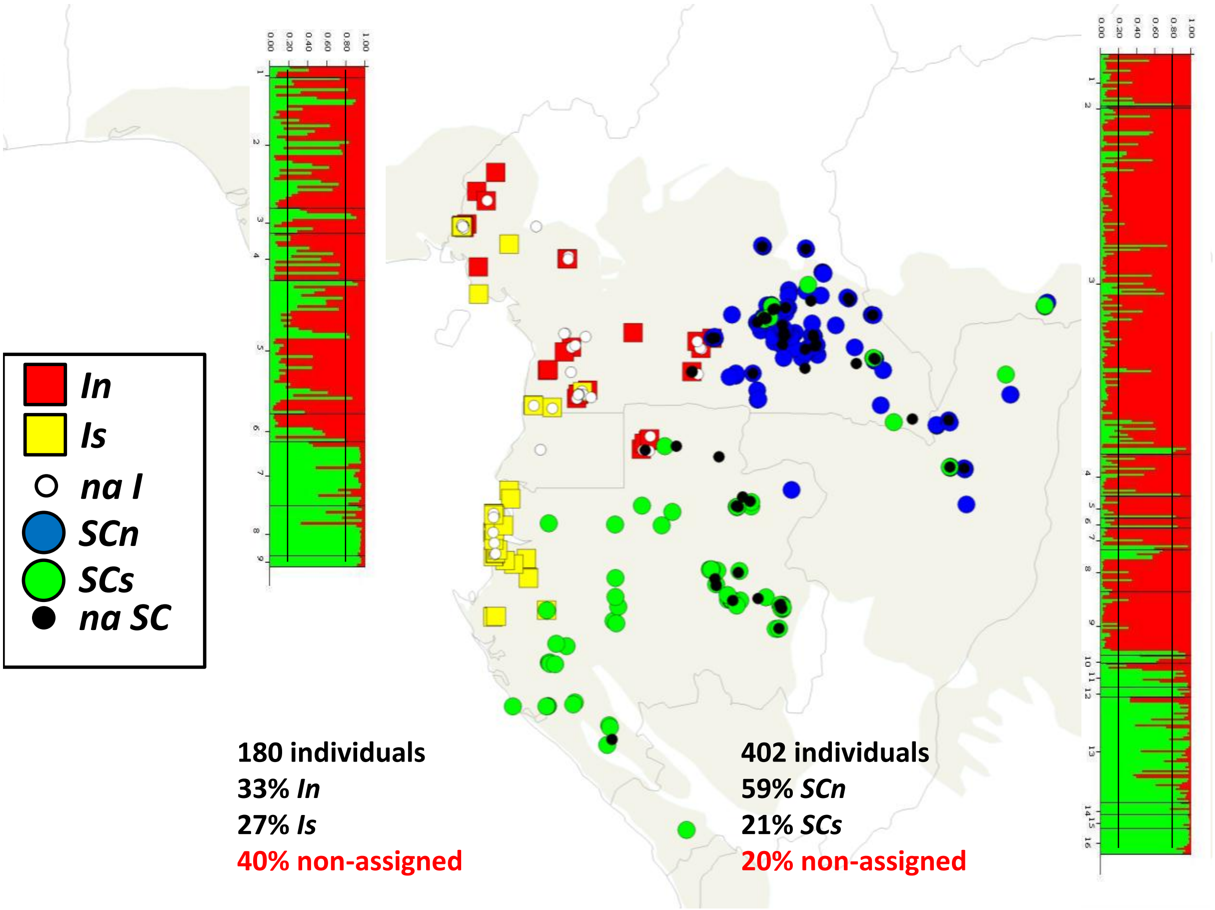

Supplement: Additional file 7 — Map of admixture zone between the Central African Northern and Southern minor gene pools in E. ivorense and E. suaveolens. [file 1471-2148-13-195-S7.pdf]
