# Supplementary material for: Large-scale pattern of genetic differentiation within African rainforest trees: insights on the roles of ecological gradients and past climate changes on the evolution of Erythrophleum spp (Fabaceae)
Source: BMC Evol Biol. 2013 Sep 12;13:195. doi: 10.1186/1471-2148-13-195 (PMC3848707; doi:10.1186/1471-2148-13-195)
Supplement: Additional file 8 — GenBank accessions numbers of the plastid DNA sequences obtained in this study. The table provides the accession numbers for all pDNA haplotypes described in this study. [file 1471-2148-13-195-S8.pdf]

Additional file 8: GenBank accessions numbers of the plastid DNA sequences obtained in this study

Gene	Species	pDNA haplotype	Individual [A]	Longitude	Latitude	Accession number
matK	*Erythrophleum chlorostachys*	H1	Australia	130.9900	-12.4900	JX840178
matK	*Erythrophleum fordii*	H2	Chine Fujian	117.4000	24.1000	JX840179
matK	*Erythrophleum africanum*	H3	EE0298	1.2069	10.2109	JX840180
matK	*Erythrophleum suaveolens*	H4	EE0463	2.2292	8.7494	JX840181
matK	*Erythrophleum ivorense*	H5	GK0543	-7.0487	5.0494	JX840182
matK	*Erythrophleum suaveolens*	H6	JD0302	13.4946	4.7498	JX840183
matK	*Erythrophleum suaveolens*	H7	LK0040	-4.9226	6.2248	JX840184
matK	*Erythrophleum ivorense*	H8	MH1793	12.4010	2.8023	JX840185
matK	*Erythrophleum suaveolens*	H9	MH1795	12.4002	2.8009	JX840186
matK	*Erythrophleum suaveolens*	H10	MH2260	1.6636	8.6208	JX840187
matK	*Erythrophleum ivorense*	H11	NB0251	10.6453	2.4523	JX840188
trnC	*Erythrophleum ivorense*	H1	CD0187	9.3383	-0.9960	JX840189
trnC	*Erythrophleum ivorense*	H2	GK0771	-9.1861	8.2322	JX840190
trnC	*Erythrophleum ivorense*	H3	EE0277	1.7500	8.9724	JX840191
trnC	*Erythrophleum ivorense*	H4	EE0279	1.7366	8.9483	JX840192
trnC	*Erythrophleum ivorense*	H5	GK0543	-7.0487	5.0494	JX840193
trnC	*Erythrophleum suaveolens*	H6	JD0589Bis	11.9355	0.4209	JX840194
trnC	*Erythrophleum suaveolens*	H7	GiD0857	12.8185	1.4852	JX840195
trnC	*Erythrophleum suaveolens*	H8	JD0585	13.8558	3.7969	JX840196
trnC	*Erythrophleum suaveolens*	H9	OH1707	13.0171	-0.7431	JX840197
trnC	*Erythrophleum suaveolens*	H10	OH1383	11.2626	4.4757	JX840198
trnC	*Erythrophleum suaveolens*	H11	OH1766	12.7724	-0.2894	JX840199
trnC	*Erythrophleum suaveolens*	H12	TOD0781	11.1604	5.7695	JX840200
trnC	*Erythrophleum suaveolens*	H13	JD0670	16.3941	1.3279	JX840201
trnC	*Erythrophleum suaveolens*	H14	GiD0851	10.1864	0.4499	JX840202
trnC	*Erythrophleum suaveolens*	H15	LK0033	-4.9341	6.2145	JX840203
trnC	*Erythrophleum suaveolens*	H16	GiD0624	11.2129	0.4319	JX840204
trnC	*Erythrophleum suaveolens*	H17	JD0587Bis	13.1474	-0.7478	JX840205
trnC	*Erythrophleum suaveolens*	H18	OH1667	10.1490	-2.3890	JX840206
trnC	*Erythrophleum suaveolens*	H19	CD0235	10.1833	-1.7097	JX840207
trnC	*Erythrophleum suaveolens*	H20	GiD1257	12.7087	-1.3408	JX840208
trnC	*Erythrophleum suaveolens*	H21	MH1791	12.4032	2.8039	JX840209
trnC	*Erythrophleum africanum*	outgroup	EE0298	1.2069	10.2109	JX840210
trnL	*Erythrophleum chlorostachys*	H1	Australia	130.9900	-12.4900	JX840211
trnL	*Erythrophleum fordii*	H2	Chine Fujian	117.4000	24.1000	JX840212
trnL	*Erythrophleum africanum*	H3	EE0298	1.2069	10.2109	JX840213
trnL	*Erythrophleum ivorense*	H4	GK0543	-7.0487	5.0494	JX840214
trnL	*Erythrophleum suaveolens*	H5	JD0302	13.4946	4.7498	JX840216
trnL	*Erythrophleum suaveolens*	H6	LK0040	-4.9226	6.2248	JX840218
trnL	*Erythrophleum suaveolens*	H7	GiD0420	10.3042	-1.4201	JX840215
trnL	*Erythrophleum suaveolens*	H8	JD0807	12.3203	-4.3090	JX840217

[A] The codes of the individuals refer to an internal database of the team “Plant population genetics and community diversity in tropical rainforests”, leaded by O. Hardy at the Université Libre de Bruxelles.
