# Supplementary material for: Large-scale pattern of genetic differentiation within African rainforest trees: insights on the roles of ecological gradients and past climate changes on the evolution of Erythrophleum spp (Fabaceae)
Source: BMC Evol Biol. 2013 Sep 12;13:195. doi: 10.1186/1471-2148-13-195 (PMC3848707; doi:10.1186/1471-2148-13-195)
Supplement: Additional file 9 — Demographic inferences obtained by MSVAR analyses at a population and gene-pool level. [file 1471-2148-13-195-S9.pdf]

Additional file 9: Demographic inferences obtained by MSVAR analyses at a population (see Additional file 1) and gene-pool level.

**Population or gene pool ID**		**Current size $N_{0\ (log)}$**	**Ancestral size N_1**	**Mutation rate μ**	**Time interval of demographic change x_a ($t_a g$)**	**θ_0 ($4N_o\mu$)**	**θ_1 ($4N_1\mu$)**	**Bayes factor [A]**	**$t_f = t_a/2N_0$**
15 (Bertoua)		0.684 [-6.067, 6.599]	4.865 [0.096, 9.633]	-4.128 [-8.989, 0.61]	2.906 [-3.522, 8.788]	-2.842 [-9.594, 0.804]	1.339 [0.821, 1.857]	44	1.921 [1.236, 2.61]
18 (Bikoula)		2.859 [-1.644, 7.284]	4.214 [-0.227, 8.703]	-3.143 [-7.601, 1.295]	5.366 [0.856, 9.922]	0.318 [-0.394, 0.953]	1.677 [1.014, 2.436]	30	2.206 [1.391, 3.008]
21 (Lastourville)		4.078 [-3.505, 12.885]	3.818 [-5.642, 11.599]	-3.463 [-8.679, 1.677]	3.256 [-9.295, 15.765]	0.957 [-8.1, 10.354]	1.217 [-7.299, 9.887]	1.6	-1.123 [-17.222, 13.123]
16 (Mindourou)		3.286 [-1.267, 7.697]	4.4 [-0.044, 8.882]	-3.49 [-7.994, 0.915]	5.294 [0.747, 9.83]	0.398 [-0.371, 1.124]	1.512 [0.929, 2.079]	35	1.707 [0.921, 2.47]
17 (Yokadouma)		1.766 [-6.411, 9.252]	4.547 [-0.985, 10.107]	-4.03 [-9.003, 0.881]	2.56 [-6.771, 11.779]	-1.662 [-10.821, 4.863]	1.119 [-1.654, 4.543]	6	0.493 [-13.038, 9.13]
25 (Yoko)		0.868 [-5.649, 6.809]	4.632 [-0.156, 9.536]	-4.194 [-8.966, 0.539]	2.727 [-3.877, 8.97]	-2.725 [-9.369, 0.944]	1.04 [0.271, 1.742]	21	1.559 [-0.723, 3.399]
"*SCn*"		3.349 [-0.559, 7.139]	4.384 [0.498, 8.214]	-3.321 [-7.124, 0.555]	5.728 [1.622, 9.557]	0.63 [0.105, 1.149]	1.666 [1.175, 2.16]	51	2.078 [1.08, 3.093]
"*SCs*"		4.168 [-3.157, 12.792]	3.758 [-6.249, 12.245]	-3.325 [-8.549, 1.917]	3.758 [-9.365, 16.274]	1.444 [-6.65, 9.918]	1.035 [-8.768, 11.142]	1.6	-0.711 [-17.383, 13.529]
5 (Pongara)		0.62 [-5.796, 6.453]	4.713 [-0.028, 9.464]	-4.2 [-9.015, 0.496]	2.846 [-3.328, 8.674]	-2.978 [-9.076, 0.686]	1.115 [0.449, 1.787]	34	1.925 [1.17, 2.736]
3 (Bipindi)		2.909 [-1.55, 7.406]	4.201 [-0.309, 8.657]	-3.189 [-7.6, 1.335]	5.178 [0.675, 9.746]	0.322 [-0.283, 0.836]	1.614 [0.979, 2.241]	28	1.968 [1.318, 2.718]
1 (Korup)		1.584 [-4.931, 7.47]	4.633 [-0.086, 9.431]	-3.85 [-8.652, 0.856]	3.334 [-3.092, 9.28]	-1.664 [-8.538, 1.283]	1.375 [0.76, 1.994]	26	1.449 [0.564, 2.449]
4 (Libreville)		-0.653 [-6.258, 4.668]	4.943 [1.421, 8.476]	-4.302 [-7.796, -0.791]	1.933 [-3.413, 7.027]	1.243 [0.7, 1.761]	-4.354 [-9.324, 0.177]	344	2.286 [1.621, 2.916]
"*In*"		4.198 [-3.062, 12.855]	4.254 [-3.195, 11.291]	-3.45 [-8.54, 1.624]	2.765 [-9.371, 14.713]	1.35 [-6.654, 9.937]	1.406 [-5.618, 9.458]	2	-1.734 [-17.325, 11.707]
"*Is*"		0.169 [-5.807, 5.802]	4.927 [0.273, 9.636]	-4.394 [-9.059, 0.29]	2.31 [-3.399, 7.887]	-3.623 [-8.996, 0.488]	1.135 [0.534, 1.731]	52	1.84 [1.116, 2.528]

[A] Posterior probability of a population decline divided by the posterior probability of a population expansion.
